# The Value of General Treatment in Diseases of Throat, Nose, and Ear

**Published:** 1899-02-18

**Authors:** 


### THE VALUE OF GENERAL TREATMENT IN DISEASES OF THROAT, NOSE, AND EAR.

At the last meeting of the British Laryngological, Rhinological, and Otological Association a discussion was held on the value of general treatment in diseases of the special organs in question. This was opened as to the pharynx and larynx by Dr. Barclay Baron, the nose and naso-pharynx by Mr. Wyatt Wingrave, and the ear by Dr. R. H. Woods. In reviewing the discussion, the president, Mr. Middlemas Hunt, said that he had constantly been struck with the improvements which had been effected by some general remedy, either in the form of medicine or a change of climate or surroundings, after he had himself failed to effect a cure. As an instance of this, he remembered the case of a little girl five or six years ago, who was suffering from laryngeal papilloma (a typical local disease to which they applied local remedies). She was a girl of five or six, and lived in the lowest slums in Liverpool, and her larynx was full of papillomata. He saw her a few times with the view of operating intra-laryngically, but she disappeared before he did so. A year ago she returned to him, she having in the meantime been taken to live with relatives in Somersetshire. The growths had during her absence shrunk so much that she could breathe comfortably, and there was little or no trace of them. However, within six months of having returned to her old surroundings in Liverpool, the growths had rapidly developed, and she died of suffocation on the way to hospital. Dr. Hunt also referred to errors which were frequently made in neurotic affections by paying too much attention to local treatment, such as the cautery, when in reality general measures were chiefly indicated. He agreed with Dr. Dundas Grant that a good many cases of nerve deafness were associated with anæmia, and were much benefited by treating the latter condition.

